# Phenotypic and Genotypic Characteristics of Methicillin-Resistant *Staphylococcus aureus* (MRSA) Related to Persistent Endovascular Infection

**DOI:** 10.3390/antibiotics8020071

**Published:** 2019-05-29

**Authors:** Liang Li, Michael R. Yeaman, Arnold S. Bayer, Yan Q. Xiong

**Affiliations:** 1 Los Angeles Biomedical Research Institute at Harbor-UCLA Medical Center, Torrance, CA 90502, USA; liangli@labiomed.org (L.L.); mryeaman@ucla.edu (M.R.Y.); abayer@labiomed.org (A.S.B.); 2 Division of Molecular Medicine, Harbor-University of California, Los Angeles (UCLA) Medical Center, Torrance, CA 90502, USA; 3 David Geffen School of Medicine at UCLA, Los Angeles, CA 90095, USA

**Keywords:** MRSA, persistence, endovascular infection, phenotype, genotype

## Abstract

Persistent methicillin-resistant *Staphylococcus aureus* (MRSA) bacteremia (PB) represents an important subset of *S. aureus* infection and correlates with poor clinical outcomes. MRSA isolates from patients with PB differ significantly from those of resolving bacteremia (RB) with regard to several in vitro phenotypic and genotypic profiles. For instance, PB strains exhibit less susceptibility to cationic host defense peptides and vancomycin (VAN) killing under in vivo-like conditions, greater damage to endothelial cells, thicker biofilm formation, altered growth rates, early activation of many global virulence regulons (e.g., *sigB*, *sarA*, *sae* and *agr*) and higher expression of purine biosynthesis genes (e.g., *purF*) than RB strains. Importantly, PB strains are significantly more resistant to VAN treatment in experimental infective endocarditis as compared to RB strains, despite similar VAN minimum inhibitory concentrations (MICs) in vitro. Here, we review relevant phenotypic and genotypic characteristics related to the PB outcome. These and future insights may improve our understanding of the specific mechanism(s) contributing to the PB outcome, and aid in the development of novel therapeutic and preventative measures against this life-threatening infection.

## 1. Introduction

*Staphylococcus aureus* is a prominent Gram-positive bacteria that can cause a wide spectrum of diseases, ranging from minor skin and skin structure infections to life-threatening diseases (e.g., bacteremia and endocarditis) [1]. In addition, *S. aureus* is a predominant cause of bacteremia and has emerged as a common entity associated with significant morbidity, complications, and mortality [2]. The emergence of methicillin-resistant *S. aureus* (MRSA) has further complicated the management of patients with staphylococcal bacteremia, increasing duration of hospital stay and cost, while decreasing the therapeutic efficacy of available antimicrobial agents [3,4,5,6]. Persistent MRSA bacteremia (PB; defined as ≥7 days of MRSA-positive blood cultures, despite appropriate antibiotic treatment) representing 15–30% of MRSA bacteremia, is especially relevant to endovascular infection and associated with high rates of morbidity and mortality (15–40%) [2,7,8]. Importantly, many PB isolates appear to be susceptible in vitro to existing gold-standard anti-MRSA antibiotics such as vancomycin (VAN) and daptomycin (DAP), based upon the Clinical and Laboratory Standards Institute (CLSI) breakpoints [7,8,9,10]. To date, we and other investigators have demonstrated specific phenotypic and genotypic features of MRSA may play important roles in the PB outcome [7,9,11,12,13,14].

In this review, we provide a summary overview of what is known about the specific genotypic and phenotypic characteristics of MRSA isolates related to the PB outcome, focusing on two distinct but interrelated facets: (1) Pathogen-drug interactions; and (2) pathogen-host interactions. In addition, salvage therapy and potential new targets to combat life-threatening MRSA outcomes are considered.

## 2. Genotypic Characteristics of MRSA Persistent Bacteremia 

### 2.1. Standard Molecular Typing

Investigations have revealed differences between PB vs. resolving MRSA bacteremia isolates (RB; defined as initial bacteremia resolved within four days of therapy) in genotypic profiles, including pulsed-field gel electrophoresis (PFGE), multi-locus sequence typing (MLST), staphylococcal protein A (*spa*) profiles, staphylococcal cassette chromosome *mec* (SCC*mec*) and accessory gene regulator (*agr*) typing, [7,9,12,15] (Table 1). Currently, PFGE is the most widely used gold standard for molecular typing of MRSA strains [16]. Fowler et al. reported that over 85% of PB isolates from one institution had closely related PFGE patterns likely representing a single genetic lineage, while only 44% of RB isolates shared the same PFGE genotyping [9]. As a DNA fingerprinting technique based upon polymorphisms within seven housekeeping genes in *S. aureus*, MLST is used to group *S. aureus* strains into unique clonal complexes (CC) [17]. Interestingly, studies focusing on the four most common CC types (e.g., CC5, CC8, CC30, and CC45) in clinical infection settings revealed that CC5 and CC30 are significantly associated with hematogenous complications of PB as compared to RB [7,18]. Another complementary genotyping methodology that appears to be useful in categorizing large *S. aureus* strain collections is *spa* typing. This technique leverages common polymorphisms within this gene locus [19]. Of great interest, an association trend of *spa* type 16 (W-G-K-A-K-A-O-M-Q-Q-Q-Q) was found among PB isolates [7,13]. SCC*mec* elements and unique genomic islands may also benefit the specific characterization of MRSA clones in epidemiological studies [20]. Among the five major SCC*mec* classes (types I-V), SCC*mec* type II appears to be associated with MRSA isolates from PB patients [7,13]. Furthermore, the *agr* operon, a quorum-sensing system in *S. aureus*, is a key global regulon that controls many critical virulence pathways in this pathogen [21]. DNA sequence polymorphisms of this locus include four *agr* groups (types I-IV) [22]. PB strains more frequently contain polymorphisms in *agr* groups II and III compared to RB strains [7,23]. Taken together, these observations suggest that specific genotypic profiles exist which differentiate PB vs. RB clinical isolates. 

### 2.2. Screening of Virulence Genes

It is well-accepted that the presence of key relevant virulent genes contributes to the PB outcome. Xiong et al. used multiplex polymerase chain reaction (PCR) to assess a panel of strategic 33 virulence genes, including regulatory-, adhesion-, exotoxin-, anti-phagocytic- and exoenzyme-encoding genes. They found PB (vs. RB) isolates to have a higher frequency of the collagen-binding protein-encoding gene (*cna*), capsular polysaccharide type 8 (*cap8*) and the toxic shock syndrome toxin-encoding gene (*tst-1*). In contrast, the cell-wall associated genes *sdrD* and *sdrE*, *cap5* and the panton-valentine leukocidin gene *pvl*, were significantly over-represented among RB vs. PB strains [7,14,18] (Table 1). While these results did not evaluate the expression of such genes, these data suggest that the presence or absence of key virulence genes could be used as a powerful tool to differentiate PB vs. RB isolates. For instance, the increased presence of adhesion gene, *cna*, might allow PB strains to exploit specific anatomic sites (e.g., endothelial substrata, bones) and potentially contribute to colonization and the persistent outcome. Besides virulence genes, a prophage (size 44.2 kb) inserted in a hypothetical protein with ostensible function involved in oxidoreduction reactions was found by whole genome sequencing in a prototype PB strain (300–169, *agr*-I, SCC*mec* IV, CC45), but absent in its RB counterpart (301–188, *agr*-I, SCC*mec* IV, CC45) [24]. The impact of prophages on virulence gene expression (e.g., *sigB*, *hld*), biofilm formation, and pathogenesis of staphylococcal infections have been reported [31,32]. For example, the β-hemolysin (*hld*) converting prophage φNM3 located in the *S. aureus* Newman chromosome, encodes modulators of innate immune responses (e.g., *sea*). An *S. aureus* Newman variant lacking φNM3 displayed organ-specific virulence defects in a murine infection model [32]. Therefore, understanding the specific role of prophage in MRSA pathogenesis overall, and PB outcomes specifically is needed. 

### 2.3. Activation of Global Regulons

The impact of global regulators (e.g., *agr RNAIII*, *sigB*, *sarA* and *sae*) on the transcription of key virulence genes and therapeutic outcomes in vivo, including the infective endocarditis (IE) model, has been previously reported [25,26,33]. In *S. aureus*, the *agr* quorum-sensing system is critical for the regulation of many virulence genes primarily through *RNA III* [12,21]. The sigma factor, SigB, represents a powerful regulator to environmental stress, especially in response to antibiotics, and to impact the expression of multiple virulence genes and global regulators, including *sarA* [34]. *sarA* has been reported to be involved in controlling many virulence genes, e.g., *hla* [21]. The *sae* two-component system was also found to be a key element in the governing the staphylococcal virulon e.g., toxic shock syndrome toxin 1 [33]. Of note, PB strains exhibited early *agr RNAIII* activation, which is significantly associated with persistence, despite VAN treatment in IE models [12,15]. Interestingly, strategic *agr* deletion in PB strains did not impact persistent outcomes [12]. These data underscored the notion that differential overall activation of the *agr* locus is not causal in PB outcomes. Therefore, factors beyond *agr* likely play important roles in PB. Recent findings revealed early on-set activation of a cadre of critical *S. aureus* global regulators, including *sigB*, *sarA* and *sae* as well as key downstream structural genes such as *cap5*, also differentiate PB vs. RB strains [25]. These genetic observations have been further verified in a representative PB (300–169) and RB (301–188) strain pair by using an in vitro cDNA microarray [25]. Importantly, these data showed that the expression of key genes involved in metabolic pathways, including nucleotide, amino acid, and carbohydrate biosynthesis was approximately 2-fold higher in PB vs. RB strains, while RB (vs. PB strains) had ≥2-fold higher expression of histidine biosynthesis and proteolysis-encoding genes [25] (Table 1). Remarkably, the significantly higher transcription of multiple purine biosynthesis pathway genes (e.g., *purF*, *purM*, *purN*, *purH* and *purl*), crucial for cell growth via DNA and RNA synthesis, was observed in PB vs. RB strains [25,35,36]. Consistently, faster growth in PB vs. RB strains in vitro has been seen in artificial media growth curve assays [25]. The activation of those global regulators (e.g., *sigB*, *sarA*, *agr*, and *sae*) and the downstream structure gene *cap5* are growth-phase-dependent [33,37,38,39], thus altered growth kinetics in distinct tissue contexts may impact the early on-set activation of global regulators in PB vs. RB strains [25] that are influenced by quorum-sensing.

## 3. Phenotypic Characteristics of MRSA Persistent Bacteremia 

### 3.1. Pathogen-Drug Interactions

#### 3.1.1. VAN Susceptibility In Vitro vs. In Vivo

VAN is established as the first-line treatment of most MRSA infections [6], as most MRSA strains are susceptible to VAN in vitro (MIC range 0.5–2.0 μg/mL) based on CLSI breakpoints. However, VAN treatment failures, especially in endovascular syndromes—due to MRSA having a VAN-susceptible phenotype in vitro—occur frequently. This persistent outcome represents a unique and important variant of traditional antibiotic resistance, and emphasizes the urgent need to better understand the specific mechanisms of this syndrome for successful treatment of such persistent infections.

#### 3.1.2. VAN-Killing Activity and Affinity for MRSA

Previous studies demonstrated that PB strains exhibited significantly higher survival than their RB counterparts on exposure to VAN under conditions of MRSA density and VAN concentrations (15 μg/mL), simulating the in vivo infection [27]. In this regard, PB strains recapitulated less susceptibility to VAN-induced bloodstream clearance vs. RB strains, which is consistent with the clinical setting [9] (Table 1). Several potential factors may contribute to this phenomenon: Firstly, it is known that VAN binds to bacterial cell-wall peptidoglycan precursor (e.g., _D_-Ala-_D_-Ala residues) to achieve its antibiotic effect [40,41]. The alteration of major cell wall components, such as wall teichoic acids and lipoteichoic acids might influence the affinity for VAN binding [41]. Abdelhady et al. found that VAN binds significantly less to PB isolates than matched RB isolates [27]. Therefore, higher survival to VAN exposure might be due to less VAN-binding capability. Secondly, as detailed above, elevated purine biosynthesis in PB strains may drive altered growth kinetics and entry into a stationary phase earlier than RB strains [25]. As adenosine triphosphate (ATP) levels begin to decrease when MRSA cells reach the stationary phase, VAN bactericidal activity would, in turn, be expected to decrease [25,39]. This model is consistent with the rapid initial growth of PB strains, leading to lower ATP levels and higher survival against VAN exposure, due to faster growth as compared to RB strains [25,39]. It is also known that a thickened cell wall appears to be associated with reduced VAN access to its active site, which leads to the reduction of VAN susceptibility [42]. However, no significant association has been seen between cell wall thickness profiles and PB strains [27].

#### 3.1.3. MRSA Biofilm Formation

It is well-known that biofilm can foster resistance to bacterial clearance mediated by antibiotics and host immune response, and bacteria protected by biofilm can serve as chronic infective foci [43]. Previous studies demonstrated that PB strains formed significantly thicker biofilm than RB strains under both static and flow conditions [27]. For instance, Seidl et al. found that, as a group, PB strains exhibited approximately 1.5-fold higher biofilm formation than matched RB strains under static conditions [11]. Moreover, under flow conditions, a representative PB strain 300–169 formed a thicker confluent biofilm (average biomass 36 µm^3^/µm^2^) than a RB counterpart strain 301–188 (0.02 µm^3^/µm^2^) [27]. Since the mechanism(s) of VAN treatment persistence related to VAN-susceptibility (based on CLSI criteria) in MRSA strains remain unclear, biofilm formation with a sub-MIC of VAN exposure was also assessed [27,44] (Table 1). Overall, significantly enhanced biofilm formation in response to a sub-MIC VAN exposure under static conditions only occurs in PB isolates, again suggesting a potential association with persistent outcomes as antibiotic exposure waxes and wanes during therapy [27,44]. 

### 3.2. Pathogen-Host Interactions

#### 3.2.1. Host Defense Peptide (HDP) Susceptibility

**Figure 1:**
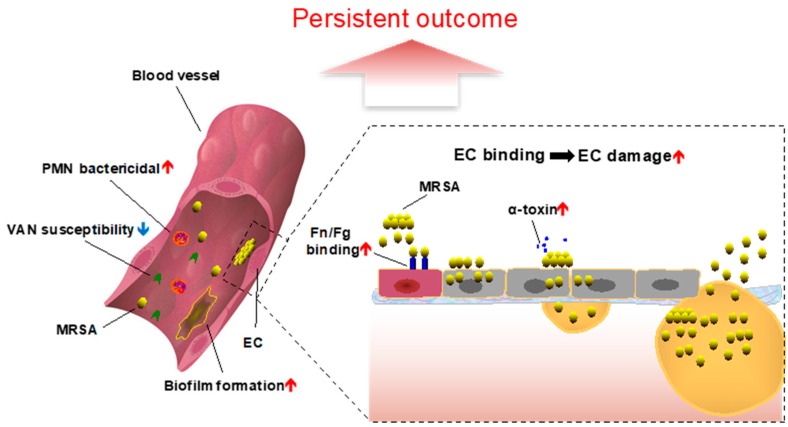
Overview of persistent bacteremia in the context of endovascular infection due to methicillin-resistant *Staphylococcus aureus* [6]. EC, endothelial cell; Fn, fibronectin; Fg, fibrinogen; PMN, neutrophils; VAN, vancomycin.

Prior to and upon entering the bloodstream, *S. aureus* must confront the essential defense mechanisms of the host, especially HDPs [45]. Such primary HDPs, including those of hematopoietic origin (e.g., neutrophils (hNP-1) and platelets (PMPs)), act in first-line host defense and often provide rapid and efficient protection against invading blood-borne pathogens, including *S. aureus* (Table 1; Figure 1) [45,46,47]. Previous studies have established that reduced susceptibility to HDPs in vitro corresponds with enhanced in vivo virulence in *S. aureus* endovascular infections [11,48]. For example, compared with RB isolates, PB strains exhibited significantly higher survival to hNP-1 and PMP exposures in vitro [7,11]. It is known that most HDPs initially target the bacterial cell membrane to initiate their lethal mechanism(s). Peschel et al. demonstrated that *mprF*, a lysyl-phosphatidylglycerol [L-PG] synthase, adds positively-charged lysine to negatively-charged PG molecules embedded in the staphylococcal cell membrane [49]. This process results in an increased net positive surface charge, thus reducing the electrostatic affinity of HDPs for the *S. aureus* cell membrane. In turn, this adaptive *S. aureus* response can lead to considerably less susceptibility to HDPs [49]. It is conceivable that such an immune subversive mechanism only occurs in vivo, contributing to the PB phenomenon during actual clinical infection. Furthermore, associations between an *mprF* variant (a single nucleotide mutation within the *mprF* gene) and persistent outcomes have been reported, supporting the view that *mprF* plays key roles in HDP susceptibility, and thus contributes to persistence [50]. Additionally, it is known that bacterial membrane fluidity can affect PMP susceptibility [28]. Bayer et al. found cationic peptide-resistant *S. aureus* (e.g., tPMP-1-resistant *S. aureus*) exhibited significantly higher degrees of membrane fluidity than susceptible counterparts [28]. Likewise, higher cell membrane fluidity was also found in PB vs. RB strains [7].

#### 3.2.2. Survival in the Face of Polymorphonuclear Leukocytes (PMNs)

PMNs provide the first line of cellular innate immunity by phagocytosing and killing bacteria [46,51]. To evade killing by PMN-mediated host innate immune system, a family of cytolytic peptides, phenol soluble modulins (*psms*) can be expressed by *S. aureus*, which effects the lysis of PMN prior to or during *S. aureus* phagocytosis [52]. Consistent with this view, our recent studies have shown PB strains exhibited significantly higher expression of *psms*, positively correlating with higher PMN lysis and survival to PMN bactericidal activity vs. RB strains (our unpublished data). Transcription of *psms* is tightly coupled to global quorum-sensing by *agr* [53]. As we have shown, the significantly earlier *agrA* and *agr RNAIII* activation in PB but not RB isolates may impact this cytotoxic capability of MRSA strains [12,15]. Taken together, the higher PMN lysis and survival to PMN bactericidal activity due to increased *psm* expression via *agr* in PB vs. RB strains likely contribute to persistent outcomes.

#### 3.2.3. Adherence to Host Endothelial Cells and Substrates

Following avoidance of host defense-mediated killing, *S. aureus* must bind to host cells and/or substrate ligands to prevent clearance by the reticuloendothelial system. In endovascular infections such as IE, *S. aureus* exploits endothelial cell (EC) adhesion and invasion to cause infection and subvert host immunity (Table 1; Figure 1) [54]. Predominant adhesins involved in *S. aureus* EC adhesion are thought to be staphylococcal cell wall-anchored fibronectin-binding proteins (FnBPs) and fibrinogen-binding protein A (clumping factor A (ClfA)), which bind to host cell-surfaces through fibrinogen (Fn) and fibronectin (Fg), respectively [55]. Interestingly, substantially higher adherence to Fn or Fg in PB isolate groups, as compared to matched RB groups, has been demonstrated [29]. Additionally, PB strains also showed stronger avidity in their binding to Fn and FnBPs, including higher adhesion force, rupture force and energy of binding vs. RB strains [29]. In contrast, there was no difference in the capacity of PB vs. RB strain in the overall adherence to ECs [7,11], suggesting specific ligand signatures as being important for EC exploitation. Overall, the higher capability of adherence to Fn and Fg or other substrates yet to be determined in PB strains (vs. RB strains) as a phenotypic feature that participates in the persistent outcome.

#### 3.2.4. Endothelial Cell Invasion and Damage 

After their successful binding, *S. aureus* can rapidly invade ECs and persist intracellularly (Table 1; Figure 1) [54]. Intracellular survival, likely in altered metabolic states (e.g., small colony variants [56,57]) may be an important factor for persistence, relapse or chronicity. These scenarios occur when internalized *S. aureus* avoids host immune defenses and effects of many antimicrobial agents [58]. In addition, over the course of long-term infection, *S. aureus* can cause cell damage to lyse the host cell and disseminate the infection (Table 1; Figure 1) [54]. In this way, the life-cycle of chronic *S. aureus* infection is perpetuated. Interestingly, our recent studies demonstrated no significant difference in the capacity of EC invasion between PB and RB study strains [30]. However, significantly greater EC damage in PB vs. RB strains was observed, which may be also associated with VAN treatment persistent outcomes in the IE model [30]. It is known that hemolysins, including α- (e.g., α-toxin), β- and δ-hemolysin, produced by *S. aureus*, are required for metastatic dissemination of infection to target organs [59]. Interestingly, higher production of α- and δ-hemolysins was found in PB vs. RB strains [9,12], but not β-hemolysin production [50]. 

#### 3.2.5. VAN Efficacy in IE Models

In the absence of antibiotics, no significant difference of MRSA densities in the target tissues (e.g., vegetation, kidney, spleen) in animals infected with PB or RB strains are observed. This finding is indicative of the view that PB and RB strains exhibited similar intrinsic virulence in the IE model [7,12,15]. However, antibiotic exposure in vivo reveals how MRSA isolates may adaptively respond to achieve PB outcomes. For example, VAN therapy resulted in significant reductions of MRSA densities in all the target tissues in animals infected with RB strains vs. untreated controls. In contrast, rabbits infected with PB strains exhibited significant resistance to VAN treatment in the IE model (Table 1) [7,12,15]. Thus, VAN treatment effectiveness in the IE model clearly distinguishes PB from RB isolates and outcomes.

## 4. Treatment Strategies for Persistent MRSA Endovascular Infection 

VAN and DAP are first-line antibiotics approved by the US Food and Drug Administration for treatment of MRSA infections [6]. To effectively treat persistent MRSA bacteremia, novel strategies are necessary, and may involve antibiotic combination therapy. Such treatment strategies might include VAN + ceftaroline (CPT), DAP + CPT, DAP + trimethoprim/sulfamethoxazole (TMP/SMX), and TMP/SMX + CPT, etc. [60]. Additionally, several case reports have been published using a protein synthesis inhibitor (e.g., linezolid) and bactericidal lipoglycopeptide (e.g., telavancin) to successfully eradicate persistent MRSA bacteremia [61,62]. As detailed above, CPT has been commonly used in combination with other antibiotics, including VAN and DAP, as salvage therapy, largely due to potent anti-biofilm activity [63]. By impairing biofilm formation as a key virulence factor in *S. aureus*, resistance to environmental stressors such as antimicrobial agents and host immune responses may also be inhibited [43]. Besides antibiotic combination, alternative strategies include a combination of small molecule compounds with existing antibiotics. Small molecule compounds that inhibit bacterial adaptive resistance in PB, alone or as adjunctive therapy combined with specific antibiotics, could conceivably restore efficacy among antibiotics presently considered ineffective. As a case in point, it is known that the major global regulator SarA controls many staphylococcal virulence factors and mediates biofilm formation via repressing core protease production [64]. Our recent studies revealed that *sarA* inactivated PB strains become exquisitely susceptible to VAN therapy in IE models [44]. In addition, *sarA* is now known to be involved in oxacillin (OXA) resistance through regulation of *mecA* expression in MRSA PB isolates [65]. This hyper-susceptibility to OXA treatment in the IE model of *sarA* mutants in MRSA strain backgrounds was also demonstrated [65]. Thus, *sarA* activation may serve important roles in the persistence of MRSA endovascular infections, and has become a new target for the development of novel therapeutic tools against these life-threatening infections. The combination therapy of SarA inhibitors (e.g., SarABI [66]) with cell wall-active antibiotics such as VAN and OXA could plausibly lead to successful treatment of persistent MRSA infections. In addition, a positive correlation between purine biosynthesis and the persistent outcomes has been recently reposted [25]. The purine biosynthesis pathway affects the activation of key global regulators (e.g., *sigB*, *sarA*, *agr* and *sae*), and VAN therapeutic efficacy in the IE model. Therefore, if selective targeting of these pathways in MRSA is possible, this approach could represent another new target for treating the persistent MRSA endovascular infection [25]. 

## 5. Conclusions

Investigating critical contributions of virulence factors and their regulation to persistent MRSA endovascular infection has revealed an overarching theme: No individual virulence determinant is sufficient to cause a persistent outcome. Furthermore, there is increasing evidence that specific, multi-variable phenotypic and genotypic signatures differentiate PB from RB in human clinical infection. Thus, differentiating the genotypic and phenotypic characteristics of MRSA strains and their adaptive responses in vitro from those that occur in the patient are urgently needed. In this way, discerning persistent vs. resolving bacteremia might be achieved by identifying biomarkers for predicting and prospectively intervening to prevent persistent outcomes. These efforts will also deepen our understanding of pathogen-drug and pathogen-host interactions in all MRSA infections. Continued studies are needed in these respects, and in novel drug development, to contribute to new and more effective agents and strategies to meet the challenge of these life-threatening infections.

## Figures and Tables

**Table 1:** Genotypic and phenotypic comparison of methicillin-resistant *Staphylococcus aureus* (MRSA) isolates from persistent bacteremia (PB) and resolving bacteremia (RB).

Characteristic	PB Isolates (Frequency)	RB Isolates (Frequency)	References
**Genotypic**			
Identical PFGE cluster	85%	44%	[9]
MLST	CC5 (53%), CC30 (21%–48%)	CC30 (18%)	[7,13,18]
*spa* type	16 (21%)	16 (18%)	[7,13]
SCC*mec* type	II (21%)	II (18%)	[7,13]
*agr* type	II (68.9%), III (21%)	II (27.8%), III (18%)	[7,23]
Screen of virulence genes	*cna* (21%), *cap8* (21%), *tst-1* (21%)	*sdrD* (47%), *sdrE* (47%), *cap5* (21%), *pvl* (56%)	[7,14,18]
Prophage	2	1	[24]
Early on-set activation of global regulons/genes in PB vs. RB	*agr RNAIII*, *sigB*, *sarA*, *sae*, *cap5*		[15,25,26]
Higher gene expression in PB vs. RB	Purine-, amino acid-, carbohydrates- biosynthesis pathway, *psms*		[25] unpublished
Lower gene expression in PB vs. RB	Histidine biosynthesis pathway genes		[25]
**Phenotypic**			
***Pathogen-Drug interactions (PB vs. RB)***			
Survival with VAN exposure	>		[27]
VAN binding	<		[27]
In vitro growth rate	>		[25]
Biofilm formation ± sub-MIC VAN (under static conditions)	>		[27]
Biofilm formation (under flow conditions)	>		[27]
***Pathogen-Host interactions (PB vs. RB)***			
Survival with HDPs exposure	>		[7,11]
Membrane fluidity	>		[7,28]
Survival with PMNs exposure	>		unpublished
Adherence to Fn and Fg	>		[29]
Fn-FnBPs binding	>		[29]
EC damage	>		[30]
α-, δ-hemolysin	>		[9,12]
VAN effectiveness in an IE model	<		[7,12,15]

NOTE. PFGE, pulsed-field gel electrophoresis; MLST, multi-locus sequence typing; CC, clonal complex; SCC*mec*, staphylococcal cassette chromosome *mec*; VAN, vancomycin; HDPs, host defense peptides; PMN, polymorphonuclear leukocytes; Fn, fibronectin; Fg, fibrinogen; EC, endothelial cell; FnBPs, Fn-binding proteins; IE, infective endocarditis; >, higher in PB vs. RB isolates; <, lower in PB vs. RB isolates.
